# The impact of DNA adenine methyltransferase knockout on the development of triclosan resistance and antibiotic cross-resistance in *Escherichia coli*

**DOI:** 10.1099/acmi.0.000178

**Published:** 2020-11-18

**Authors:** Lewis Hughes, Wayne Roberts, Donna Johnson

**Affiliations:** ^1^​ Biomedical Sciences, Leeds Beckett University, Leeds, UK

**Keywords:** DNA adenine methyltransferase, triclosan, antibiotic resistance, antimicrobial resistance

## Abstract

**Background:**

DNA adenine methyltransferase (*dam*) has been well documented for its role in regulation of replication, mismatch repair and transposition. Recent studies have also suggested a role for *dam* in protection against antibiotic stress, although this is not yet fully defined. We therefore evaluated the role of *dam* in the development of antibiotic resistance and triclosan-associated cross-resistance.

**Results:**

A significant impact on growth rate was seen in the *dam* knockout compared to the parental strain. Known triclosan resistance-associated mutations in *fabI* were seen regardless of *dam* status, with an additional mutation in *lrhA* seen in the *dam* knockout. The expression of multiple antibiotic resistance-associated genes was significantly different between the parent and *dam* knockout post-resistance induction. Reversion rate assays showed that resistance mechanisms were stable.

**Conclusions:**

*dam* knockout had a significant effect on growth, but its role in the development of antibiotic resistance is likely confined to those antibiotics using *acrAD*-containing efflux pumps.

## Data Summary

The whole-genome sequencing data of the strains used in this study are available from NCBI under the BioProject accession number PRJNA517874.

## Background

In order to maximize survival, clonal bacterial populations (cultured from a single colony) exhibit phenotypic cell–cell variation. While it has generally been assumed that mutation, spread through the population via vertical descent, is the cause of such variation, it is becoming increasingly apparent that epigenetic changes are also involved [1, 2]. Adenine methylation is the most common epigenetic change in prokaryotes [3], and in Gram-negative bacteria it is primarily mediated by DNA adenine methyltransferase (*dam*) [3, 4]. *dam* has been shown to be involved in mismatch repair [3, 4], regulation of replication [5, 6], transposition [7, 8] and control of gene expression [9]. *dam* has also been linked to antibiotic resistance (ABR) – Adam *et al*. saw increased resistance to ampicillin, tetracycline and nalidixic acid in *Escherichia coli* as a result of epigenetically induced changes in the expression of resistance-associated genes [2]. Conversely, a role for *dam* has been suggested in protection against antibiotic stress; *E. coli* lacking *dam* exhibit compromised survival in the presence of ampicillin, likely as a result of a build-up of double strand breaks [10]. The expression of broad-spectrum resistance-associated genes such as the *acrAB/D-tolC* efflux pumps of *E. coli* have been shown to be regulated by *dam* [11–13], adding support to the potential role for *dam* in the development of ABR. Given that *dam* homologues are widespread amongst bacteria [14], a full understanding of the role of adenine methylation in the development of resistance is critical for the identification of potential new targets for drug development.

Triclosan (TCS) is a broad-spectrum biocide that has recently been restricted due to concerns that it may have toxic or carcinogenic effects, in addition to concerns about antibiotic cross-resistance [15–18], but it is still used in a range of products such as soaps and deodorants [19]. In *Salmonella enterica* serovar Typhimurium, TCS selects for increased resistance to ampicillin, tetracycline, ciprofloxacin and kanamycin, and also increased expression of the *acrAB* efflux pump [20, 21]. Furthermore, TCS has been seen to modulate efflux pump expression directly in *Stenotrophomonas maltophilia *, by binding to the repressor s*meT*, allowing expression of the s*meDEF* efflux pump [22]. While data [23] support the involvement of efflux pumps in TCS-mediated cross-resistance, the specific mechanisms have yet to be fully elucidated. Due to the heavy commercial dependence on TCS, the Scientific Committee in Consumer Safety highlights the need for further *in vitro* studies to demonstrate if, when used at sub-lethal concentrations, TCS causes the development of antibiotic cross-resistance and to determine the mechanisms behind this [15]. Our hypothesis is that *dam* is able to regulate efflux pump expression and that this mechanism underpins the development of TCS-induced cross-resistance.

## Methods

### TCS resistance

The minimum inhibitory concentration (MIC) of the parental and *dam* (ECK3374) knockout (* E. coli* BW25113 strain and isogenic knockout strain, Keio Knock-out Collection, Dharmacon) was determined using broth microdilution. Parallel *dam* knockout and parental cultures were serially sub-cultured in nutrient broth with increasing TCS concentrations for 7 consecutive days. TCS was used at 1 µg ml^−1^ until day 5 and 10 µg ml^−1^ between days 5 and 7. The growth rates of initial cultures and TCS-resistant mutants, obtained from single colonies cultured on nutrient agar (10 µg ml^−1^ TCS), were assessed over 24 h using spectrophotometry and antibiotic cross-resistance using disc diffusion (MASTRING-S systemic Gram negative M14 multi-disc, MAST, UK). The sensitivity of each strain was determined according to the guidelines in the BSAC Methods for Antimicrobial Susceptibility Testing [24]. Fitness costs were calculated from relative growth rates.

### RT-qPCR

RNA was extracted from starting cultures and resistant mutants, using the PureLink RNA Mini kit (Thermo Fisher Scientific, UK) following the standard protocol. RNA concentration and 260/230 and 260/280 ratios were determined through microvolume spectrophotometry (Denovix). RNA integrity was assessed via gel electrophoresis. Non-degraded samples (260/230~2.2 and 260/280~2.0) were accepted for cDNA synthesis using the Verso cDNA Synthesis kit (Thermo Scientific) following the standard protocol. RTq-PCR was performed with iTaq universal SYBR Green supermix (Thermo Fisher, UK) using a CFX96 Touch Real-Time PCR Detection System. The primer sequences were as indicated in Table 1. Cycling conditions were as follows: 95 °C for 5 min; 40 cycles of 95 °C for 5 s; 60 °C for 30 s. *hcaT* was shown to be a suitable reference gene by Normfinder, as determined experimentally from three genes (*hcaT, cysG* and *rpoS*) [25]. Fold change was calculated using ΔΔCt and was relative to the starting parent strain. Differences in mean fold changes were assessed using Welch’s analysis of variance (ANOVA) with a significance level 0.05 in SPSS (V.25).

**Table 1.** RT-qPCR primer sequences

Gene	Primer sequence		Gene	Primer sequence	
*acrA*	F	GAGTACGATCAGGCTCTGGC	CysG	F	TTGTCGGCGGTGGTGATGTC
	R	AGGAAGTCGTTGCTGGACTG		R	ATGCGGTGAACTGTGGAATAAACG
*acrB*	F	CAGGATCAACGCCACCAGTA	*rpoS*	F	TATGAGTCAGAATACGCTG
	R	AGGAAGTCGTTGCTGGACTG		R	GGAACAGCGCTTCGATATT
*acrR*	F	AAGAAACGCGCCAACACATC	HcaT	F	GGCACTGCTGACACTTCTCT
	R	CAGCGAGGTGGATGATACCA		R	TAGTGACCAGTTTGCCCGTC
*tolC*	F	CGTTTTTCGGCTTCTTTCAG	*lrhA*	F	GGCGGTAAGCCATCTACTCC
	R	TTTTAACGGGCCTGGTAG		R	CCTCGCCAACACACTGGTACT
*marA*	F	CATAGCATTTTGGACTGGAT	Fabl	F	CCGCGTAGAAGAATTTGCCG
	R	TACTTTCCTTCAGCTTTTGC		R	GATCGGACCAGCAGAGATG
*marR*	F	AGCGATCTGTTCAATGAAT			
	R	TTCAGTTCAACCGGAGTAAT			

### Reversion rate assay and ability to grow at high TCS concentrations

Initial strains and TCS-resistant mutants were cultured in TCS-free nutrient broth for 24 h at 37 °C. Ability to grow in the presence of TCS was assessed by plating 100 μl of the overnight culture on nutrient agar/TCS plates (10 µg ml^−1^). Cultures were then propagated in TCS-free media every 24 h for 10 days with a sample being plated on nutrient agar/TCS plates alongside. The reversion rate was determined as the time in days until the loss of TCS resistance. As reversion was not noted after 10 days, we continued to assay the upper limit of resistance. Therefore, at days 10, 15, 20, 25 and 30 TCS concentration in the plates was increased to 0.1, 0.15, 0.2, 0.5 and 1 mg ml^−1^, respectively.

### Genome sequencing

DNA was extracted using a PureLink Genomic DNA Extraction kit (Invitrogen) with the standard protocol. A microvolume spectrophotometer (Denovix) was used to quantify the concentration and 260/230 and 260/280 ratios.

Initial cultures and resistant mutants of the parent and *dam* knockout strains were sequenced using Illumina MiSeq chemistry and 2×250 bp paired end reads (MicrobesNG, UK). Raw reads were processed using the Comprehensive Genome Analysis pipeline in PATRIC and variants identified using the Variation Analysis service [26]. The BioProject accession number for the sequences is PRJNA517874.

## Results

### *Dam* loss had a significant effect on the generation time of *E. coli*

*Dam* has been linked to alterations in the growth of *E. coli* [27] and, given this, we initially sought to confirm these findings by assessing the baseline effect of *dam* on growth and in doing so confirm that alterations post-TCS resistance were not linked to significant differences pre-induction. Both strains were able to grow, confirming the non-essential status of *dam* in *E. coli *, but the mean generation time for the *dam* knockout was significantly higher compared to the parent pre-TCS exposure. The absence of *dam* was seen to equate to a fitness cost of −7.4 % (Table 2). There was an increased generation time for the parental strain, and this is believed to be related to the culture volume (100 µl) and slightly decreased aeration from the shaking of the spectrophotometer.

**Table 2.** Mean generation times and fitness costs associated with *dam* loss and TCS resistance

	Mean generation t ime+/−se min	Fitness cost relative to parent start+/−se %
**Parent start**	66.2+/−0.4	–
**TCS-resistant parent**	81.9+/−1.2	−23.5+/−1.9
***dam* knockout start**	72.0+/−0.6	−7.4+/−1.1
**TCS-resistant *dam* knockout**	81.5+/−2.0	−22.9+/−3.2

### *Dam* knockout induced resistance to TCS and altered global antibiotic resistance patterns

**Fig. 1.**
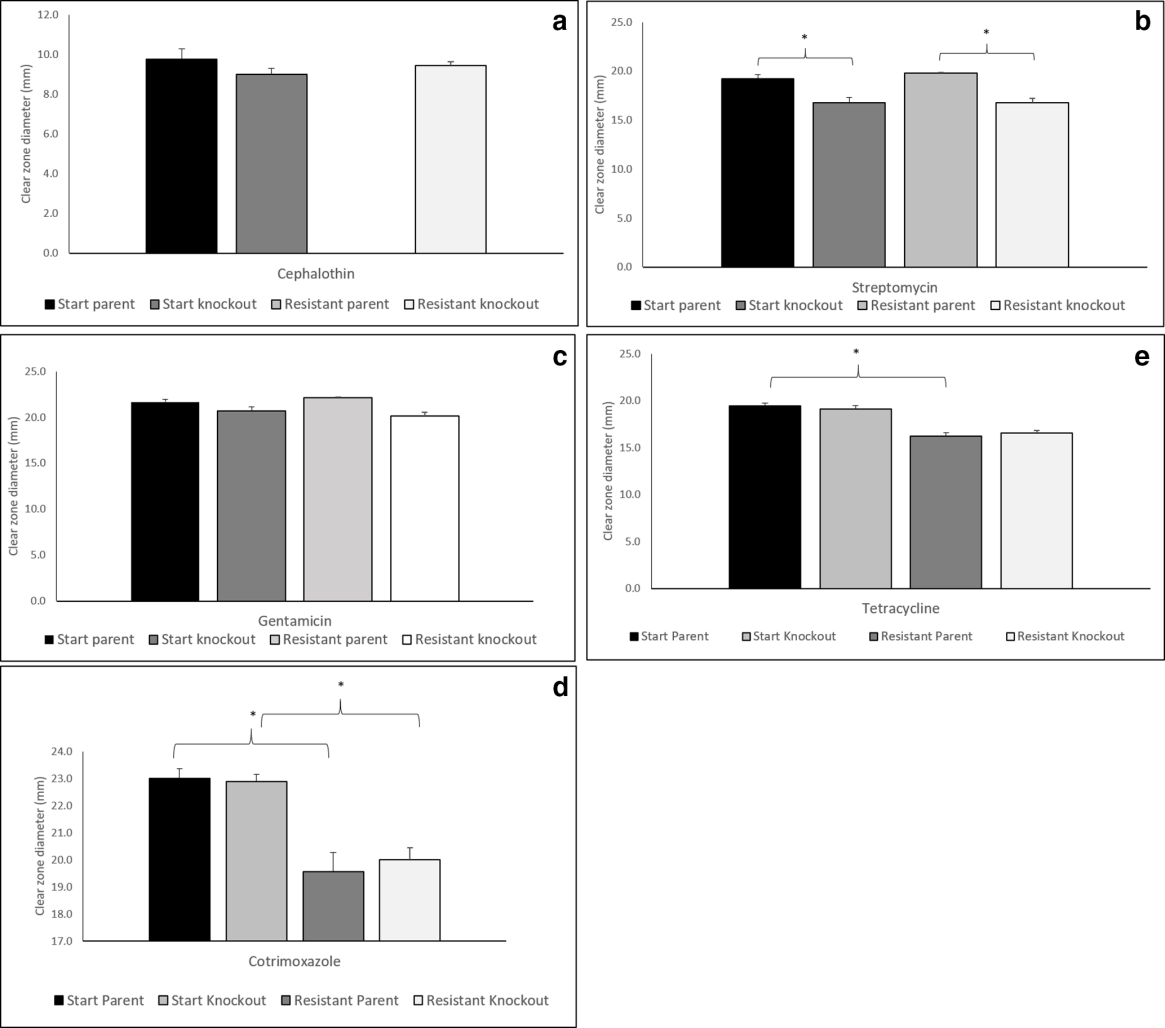
Cross-resistance to cephalothin developed alongside TCS resistance in the presence of *dam* (a), but the loss of *dam* also contributed to increased resistance to streptomycin (b) and gentamicin (c). The TCS-resistant parent was fully cross-resistant to cephalothin (zone of 0 mm). TCS resistance induced increased resistance to tetracycline in the resistant parent compared to the start parent (d). Decreased resistance to cotrimoxazole was observed in the start parent compared to the TCS-resistant parent and the start knockout compared to the TCS-resistant knockout (e). Diameters were calculated from three repeats of three lines (three technical repeats of three biological repeats, *n*=9), error bars show standard error, * denotes *t*-test *P*<0.05.

Since methylation of GATC sites by *dam* mediates survival of *E. coli* in the presence of antibiotics [10], we assessed the impact of *dam* knockout on a range of antibiotics (ampicillin, cephalothin, colistin sulphate, gentamicin, streptomycin, sulphatriad, tetracycline, cotrimoxazole). Prior to the induction of TCS resistance, and with the exception of streptomycin (Fig. 1b), the resistance profiles of the *dam* knockout and the parent were not significantly different. Within the parental strain, cross-resistance to cephalothin developed alongside TCS resistance (Fig. 1a). However, within the *dam* knockout the development of TCS resistance led to increased resistance to streptomycin and gentamicin (Fig. 1b, c), suggesting that the loss of *dam* may have a role in resistance to aminoglycoside antibiotics. In contrast, the increase in resistance to cotrimoxazole (Fig. 1e) for both the TCS-resistant parent and the *dam* knockout suggests a non-*dam*-dependent mechanism of cross-resistance. Additionally, the *dam* knockout was also more resistant to TCS, with an MIC of 0.9 μg ml^−1^ compared to 0.4 μg ml^−1^ for the parent, suggesting a further role for *dam* in resistance to TCS.

### The *dam* knockout showed no difference in the mechanism of TCS resistance or in the development of resistance-associated mutations

Loss of *dam* has been associated with an increase in mutation rate through the partial induction of the SOS regulon and loss of mismatch repair capability, suggesting the possibility of increased genomic instability [28]. We hypothesized that this increase in mutation rates could result in global antibiotic resistance-associated mutations within the TCS-resistant *dam* knockout. In order to assess this, we sequenced parental and *dam* knockout strains pre- and post-TCS exposure (Table 3). Sequencing showed that there were few mutations, with the most significant being a substitution present in the *fabI* gene, resulting in a change at amino acid 93 (glycine to valine). This mutation has been widely associated with TCS resistance and confers altered binding properties to enoyl-acyl carrier protein reductase (ENR) [29].

**Table 3.** Variants identified in the starting knockout strain and the TCS-resistant mutants

	Mutation	Gene	Mutation	Amino acid change	Position	Fraction of sequences
**TCS-resistant parent**	Nonsyn	*fabI*	278G>T	Gly93Val	1 345 019	1
	Nonsyn	*tfaD*	CAGCGAC>TAACGAT	GlySerAsp2GlyAsnAsp	577 016	0.57
	Synon	*tfaD*	18C>A	Ile6Ile	577 004	0.53
	Synon	*yecE*	54G>T	Gly18Gly	1 945 705	0.52
	Intergenic	*tfa-nu1*	AC >GT	–	576 968	0.52
	Intergenic	*tfa-nu1*	C>G	–	576 974	0.56
	Intergenic	*tfa-nu1*	GCGGGCC>ACGCGCG	–	576 980	0.6
***dam* knockout start**	Intergenic	*kgtP-5SrRNA (rrnG operon*)	T>C	–	2 719 426	1
**TCS-resistant *dam* knockout**	Nonsyn	*fabI*	278G>T	Gly93Val	1 345 019	1
	Intergenic	*fabI*	G>A	–	1 345 411	0.51
	Synon	*pcnB*	243G>A	Val81Val	155 338	0.95
	Insertion	*lrhA*	28_29insACCTCG	Asn10_Leu11insLeuAsp	2 400 079	0.86
	Intergenic	*kgtP-5SrRNA (rrnG operon*)	T>C	–	2 719 426	1

In the TCS-resistant *dam* knockout there was an additional mutation upstream of *fabI* as well as upstream of 5 s rRNA, and an insertion in *lrhA*, a transcriptional repressor of the *lysR* family. Within the resistant parent there were no other mutations commonly associated with broad-spectrum AMR, and TCS resistance was therefore attributed to those mutations seen within *fabI*. For both the TCS-resistant parent and the *dam* knockout, these mutations were seen to be highly stable, as neither the parent nor the *dam* knockout TCS-resistant mutants reverted to sensitivity after 30 days of growth in the presence of TCS at concentrations up to 100 times greater than the pre-TCS resistance MIC.

### There were significant differences in the expression of resistance-associated regulatory genes in the *dam* knockout

**Fig. 2.**
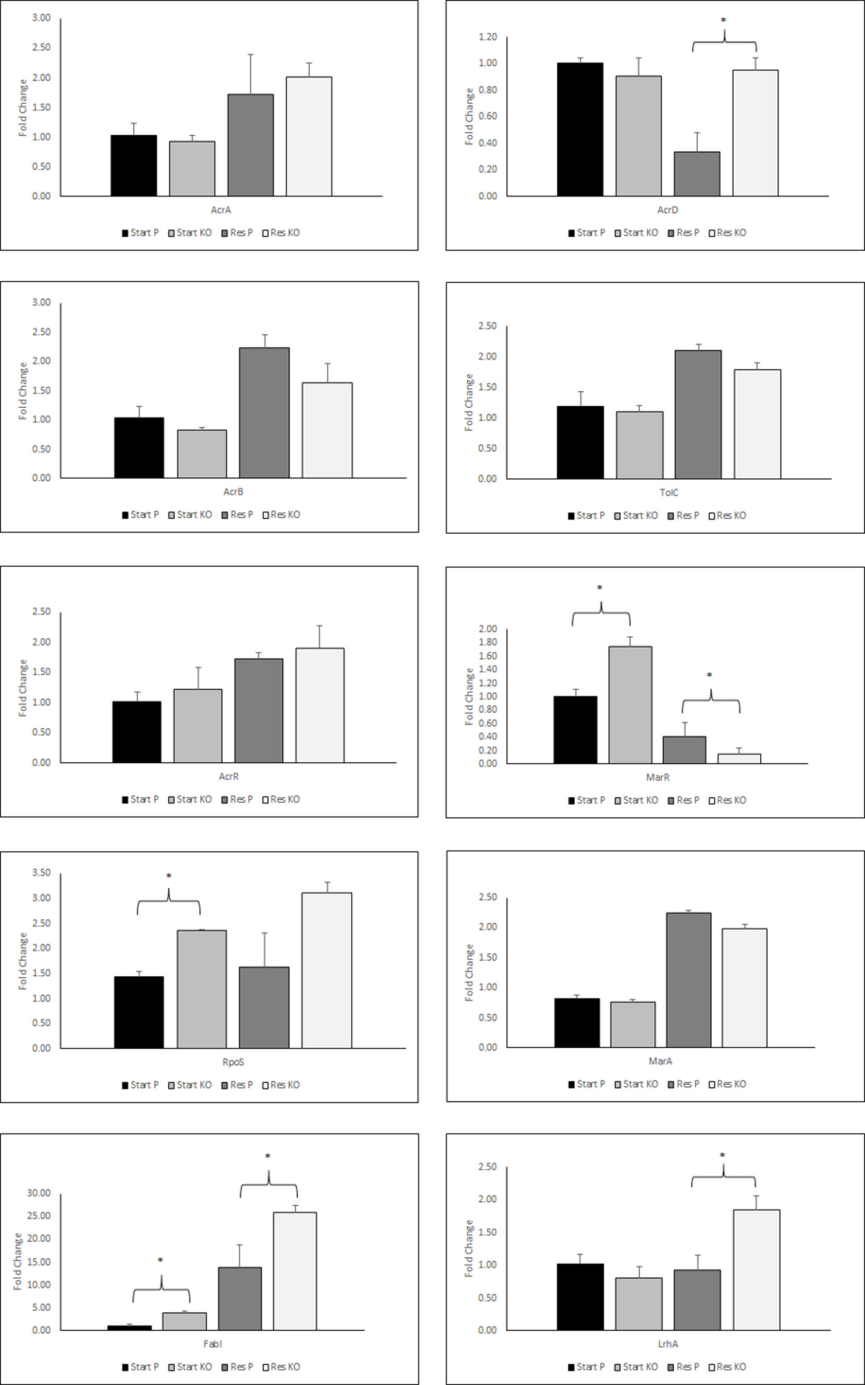
Expression of the components of the *acrAB-tolC* efflux pump was not significantly different in the *dam* knockout compared to the parent, but expression was increased in both TCS-resistant strains. There were significant differences between the *dam* knockout and parent pre-resistance induction in *rpoS*, *fabI* and m*arR*. In TCS-resistant strains, there were only significant differences in the expression of a*crD*, *lrhA*, *fabI* and *marR*, although there were significant differences between pre-post-induction levels of *marA* for both the parent and *dam* knockout. Expression was calculated from three repeats of three lines (three technical repeats of three biological repeats, *n*=9), error bars show standard error, * denotes *t*-test *P*<0.05.

Several resistance mechanisms are mediated through changes in efflux pump expression, and TCS-associated cross-resistance has been suggested to act via efflux pumps [23, 30]. In order to assess both these observations, we investigated the expression levels of several efflux components. We also looked at the expression levels of genes whose sequences were mutated (Fig. 2). Interestingly, post-resistance induction, we found no significant differences between the parent and *dam* knockout in expression of *acrAB-tolc* (Fig. 2a, c and d), or within the multiple-antibiotic resistance protein *marA* (Fig. 2h), or the transcriptional regulator *acrR* (Fig. 2e), suggesting that the action of these pathways is not *dam*-dependent. In contrast, expression levels differed for a*crD*, *marR*, *rpoS*, *fabI* and *lrhA* (Fig. 2b, f, g, i and j)*,* suggesting that *dam* affects the regulation of some efflux pump genes and that TCS-associated antibiotic cross-resistance may be more predominant for antibiotics whose mechanisms of resistance are related to efflux by the *acrAD-tolC* efflux pump, such as some aminoglycosides [31], an observation supported by increased resistance to streptomycin and gentamicin in the TCS-resistant *dam* knockout. The elevated expression levels of *rpoS* seen within the *dam* knockout (Fig. 2g may account for the relatively few mutations seen within the start and the TCS-resistant *dam* knockout, as *rpoS* has a protective role in DNA damage due to its ability to upregulate both the SOS response and DNA polymerase Pol II.

## Discussion

Methylation of the adenine within 5′-GATC-3′ sites of double-stranded DNA following replication is a key process within DNA mismatch repair [32], alterations in gene expression [12] and the initiation of chromosome replication [33] and, as such, loss of *dam* has potentially wide-reaching effects. Here we have shown that the loss of *dam* results in a significantly decreased mean generation time and that its absence contributes to differences in the ABR profile compared to a wild-type parental strain and confers a measure of resistance to the antimicrobial TCS. The increased generation time (Table 2) seen with the *dam* knockout matches observations of *dam*-deficient strains of uropathogenic *E. coli* [34]. This increase is likely a consequence of the role of *dam* in replication [32, 34]. We also observed a longer lag period in the *dam* knockout, which may be a consequence of the lack of replication initiation coordination. Whilst a clear fitness cost is seen within the *dam* knockout (Table 2), this value is significantly less than that for the TCS resistance *dam* knockout and parent. We also did not detect any inherent mutations, expression values or antibiotic susceptibilities within the starting *dam* knockout that would indicate that it is inherently unable to develop resistance. A comparison of the ABR profiles (Fig. 1) between the parent and *dam* knockout strains prior to resistance induction showed that the *dam* knockout is marginally more resistant to the tested antibiotics than the parent strain, although the difference is only significant for streptomycin. We attribute this increase in resistance to the observed increased expression of *rpoS,* a general stress response regulator [35], which is significantly higher in the starting *dam* knockout (Fig. 2g). A recent study has shown that 100 genes are regulated by *rpoS* in *E. coli *, including penicillin-binding proteins (PBPs) [36]. *rpoS* has been associated with antibiotic resistance to β-lactams in *E. coli* [37] and tolerance to carbapenems in *Pseudomonas aeruginosa* via regulation of PBP expression [38], and been demonstrated to have a significant effect in single- and double-strand DNA break repair and tolerance [39]. *rpoS* has also been shown to be protective against the type of DNA damage caused by aminoglycosides in *E. coli* [40]. This mechanism was not exacerbated by TCS resistance within either the parent or DAM knockout (Fig. 1b), demonstrating that this is not a mechanism of cross-resistance and nor is it DAM-dependent here. The increased *rpoS* expression may also explain the low number of mutations seen within the TCS-resistant *dam* knockout, as *rpoS* has a protective role in DNA damage due to its ability to upregulate the ada response [37]. Cross-resistance to cephalothin was seen within the TCS-resistant parent but not the TCS-resistant knockout, suggesting a role for *dam*. Broadly speaking, β-lactam resistance occurs via one of two mechanisms, either through the production of β-lactamase, which is most common in Gram-negative species, or via the production of an altered penicillin-binding protein [41]. As neither of these pathways would derive from TCS resistance, we suggest that cephalothin cross-resistance developed from upregulation of *marA* and the concurrent decrease of *marR* (Fig. 2f, h), whereby upregulation of the resistance-nodulation-cell division (RND) family efflux systems (*acrAB*, *acrAD*, *acrEF*, *mdtEF* and *mdtABC*) results in resistance. Significantly, each of the five listed RND family drug exporters have been shown to confer resistance to β-lactam antibiotics within *E.coli* [42]. Whilst significant differences in the expression of the *acrAB-tolC* system were not seen, it is possible that *mdtEF* and m*dtABC* expression levels were elevated.

Cross-resistance to tetracycline was increased in the TCS-resistant parent and *dam* knockout. Within the TCS-resistant parent we saw no mutations within the ribosomal-binding site, or chromosomal mutations leading to increased expression of the intrinsic resistance tetracycline tet-on tet-off system (Table 3), suggesting that the mechanism of resistance is broad-spectrum efflux by an unobserved mechanism such as that highlighted above. Additionally, we observed the development of cross-resistance to cotrimoxazole for both the TCS-resistant parent and *dam* knockout. In *E. coli,* cotrimoxazole resistance is primarily via mutations in the target sites of the two composite drugs trimethoprim [dihydrofolate reductase (*dfr*)] and sulphonamides [dihydropteroate synthase (*folP*)] [43, 44]. However, we did not detect any point mutations in either of these genes (Table 3). Increased expression of *E. coli acrAB-tolC* and *mexAB-oprM* systems have been shown to confer resistance to sulphonamides. Efflux of sulphonamides would inevitably reduce the overall effectiveness of cotrimoxazole, as sulphonamides and trimethoprim work bactericidally in combination to reduce cellular tetrahydrofolic acid levels. As discussed previously, expression of the *acrAB-tolC* genes did not increase post-TCS resistance, and resistance may therefore be mediated by an alternative efflux system.

An interesting observation from this work is the identification of the insertion mutation in l*rhA* in the *dam* knockout (Table 3). This mutation is also seen in chloramphenicol-resistant *dam* knockouts (Hughes, *et al*., unpublished data). This mutation causes an inframe insertion of leucine and aspartic acid. While the effect of the mutation is unknown, it is predicted to be deleterious by the Protein Variation Effect Analyzer (Provean) [45]. *LrhA* belongs to the *lysR* family and contains a helix–turn–helix (HTH) DNA-binding domain (amino acids 11–68), which overlaps with the insertion mutation (between amino acids 10 and 11). Mutations in such domains in other HTH-containing DNA-binding proteins have been shown to decrease DNA-binding capability [46, 47]. If the DNA-binding ability of *lrhA* is decreased as a consequence of this mutation it would no longer be able to repress *rpoS* to the same extent as the wild-type, which may contribute to the increased *rpoS* expression seen here. Within the TCS-resistant *dam* knockout a synonymous mutation was seen in *pcnB*. While deletion mutations of *pcnB* have been shown to confer resistance to high concentrations of chloramphenicol, ampicillin and kanamycin, the significance of this mutation, apart from a general contribution to altered fitness costs, is unknown [48]

Marginally increased resistance to TCS was seen in the *dam* knockout strain compared to the parental strain prior to resistance induction, with MICs of 0.9 μg ml^−1^ v 0.4 μg ml^−1^, respectively. Post-TCS resistance, TCS-exposed knockouts and parent strains were able to grow at concentrations up to 100 times greater than the initial concentration. TCS acts by disrupting the synthesis of fatty acids by competitive inhibition of ENR. TCS interaction increases the affinity of ENR for nicotinamide adenine dinucleotide (NAD+), resulting in the development of the stable ternary complex ENR/–NAD/TCS. In this form, ENR is unable to synthesize fatty acids [47]. In the *dam* knockout, the level of *fabI* is significantly increased (Fig. 2. I), and this may contribute to a higher tolerance for TCS through the increased availability of ENR. There is an additional mutation (G>A) upstream of *fabI*, (Table 3); this base is the first site of the f*adR*-binding site located in the *fabI* promoter [49]. *FadR* is a transcriptional activator of fatty acid synthesis and its loss has been shown to significantly decrease *fabI* expression [50]. While the functional effect of this mutation is unknown, it may be that it results in increased binding of *fadR* to the *fabI* promoter and so contributes to the increased expression seen here. Increased *fabI* expression is also seen in the resistant *dam* knockout and parent, with a significantly greater level of expression seen in the *dam* knockout, which may explain the shorter time to resistance observed here – 3 and 5 days for the *dam* knockout and parent, respectively. The resistance-associated mutation, Gly93Val, was seen in all sequences for both the resistant parent and *dam* knockout. This mutation is associated with changes to the protein structure and altered interactions with TCS leading to significant increases in resistance [29]. This is reflected by the ability of the post-resistance induction strains to grow in the presence of a 500-fold greater concentration of TCS (450 μg ml^−1^).

While *dam* plays an important role in a range of key physiological processes, and loss of its activity confers a measure of inherent resistance to TCS, the loss of *dam* does not appear to enhance the development of cross-resistance in most cases, either through an increase in the number of mutations or in the expression level of efflux associated. These findings match the assertion of Cohen *et al*. [10] that *dam* provides structural support during exposure to antibiotics. This may, however, depend on the specific mechanism of the agent investigated (e.g. antibiotics whose resistance mechanisms rely on non-*acrAB-tolC* efflux or antibiotics that target DNA replication, such as quinolones) [10].
